# Parenting Style and Their Influence on Children’s Dental Health in Riyadh, Saudi Arabia: A Cross-Sectional Study

**DOI:** 10.3390/children13030316

**Published:** 2026-02-24

**Authors:** Areej Alsiwat, George Kitsaras, Anne-Marie Glenny, Haya Alayadi, Michaela Goodwin

**Affiliations:** 1 Division of Dentistry, School of Medical Science, Faculty of Biology, Medicine and Health, University of Manchester, Manchester M13 9PL, UK; georgios.kitsaras@manchester.ac.uk (G.K.); a.glenny@manchester.ac.uk (A.-M.G.); 2 Dental Health Department, College of Applied Medical Sciences, King Saud University, P.O. Box 10219, Riyadh 11433, Saudi Arabia; halayadi@ksu.edu.sa

**Keywords:** parenting, parental style, parent–child relation, dental caries, oral health

## Abstract

**Background:** Families, including mothers and fathers, create a safe, stable environment if they work together in setting rules for their children. Research on parenting style among Saudi Arabian parents in Riyadh is lacking. Analyzing and assessing these factors will facilitate the formulation of evidence-based methods to enhance parenting practices, which is the main aim of this study. **Methodology:** This cross-sectional study conducted in Riyadh, Saudi Arabia, explored the parenting style of primary school parents using a validated and widely used questionnaire. Clinical examination for the prevalence of children’s dental caries was performed according to World Health Organization (WHO) criteria. Additionally, parents were asked to complete a structured questionnaire to evaluate their child’s oral health by using the WHO’s Oral Health Questionnaire for Children. **Results:** In the present study, the survey was completed by 506 parents from 14 schools and the authoritative parenting style emerged as the most common among participants, with 88.7% for mothers and 81.4% for fathers. The findings indicated there was no evidence of an association between mothers’ and fathers’ authoritative parenting style with the prevalence of dental caries [OR = 1.021, 95% CI: 0.79, 1.31, *p* = 0.871], [OR = 0.940, 95% CI: 0.71, 1.22, *p* = 0.653], respectively. However, there was evidence of an association between mothers’ authoritative parenting style and higher fruit consumption [OR = 1.27, 95% CI: 1.028, 1.570, *p* = 0.027], meaning fruit consumption increased with higher authoritative style scores. **Conclusions:** The present study indicated that the authoritative parenting style was the most often reported among participants. This study found no evidence of an association between parenting style and the prevalence of dental caries or brushing frequency; however, a difference in fruit and vegetable consumption was seen, with increased intake linked to mothers’ authoritative parenting style.

## 1. Background

One of the most widely recognized theories on parenting styles is Baumrind’s approach, which states that consistent parenting styles are formed through a collection of behaviors and that it is preferable to examine general patterns rather than isolated acts. Her initial classification identified three main parenting styles: authoritative parents are kind and accommodating while also establishing clear guidelines and expectations; authoritarian parenting is marked by strictness and a lack of warmth; and permissive parents are affectionate but do not enforce structure or boundaries [1].

Later, in the 1980s, researchers Maccoby and Martin expanded Baumrind’s framework. They proposed that parenting styles could be described along two dimensions: demandingness (the degree to which parents enforce rules and expectations) and responsiveness (the level of warmth and support provided). Based on these traits, they defined four parenting styles, which can be summarized as follows:

- Authoritative: high support and high expectations
- Authoritarian: low support, high expectations
- Indulgent (permissive): high support, low expectations
- Neglectful: low support and low expectations [1]

The influence of culture on parenting styles has been the subject of many studies. One study found that authoritarian parenting, based on parent reports, was linked to more behavioral and emotional problems among children in neighborhoods with higher proportions of Asian families. In contrast, low-income families were less likely to use authoritarian parenting, which was associated with fewer behavioral problems such as children being disruptive, aggressive, or defiant [2]. Research has pointed out that mothers and fathers tend to think differently when thinking about what causes success in parenting. Mothers are believed to influence their children more than fathers. However, the difference between mothers and fathers in their attitudes and beliefs has not yet been examined in different cultures [3]. Culture may have an impact on how mothers and fathers raise their children. For example, the expression “strict father, kind mother” describes the assumption in many Asian cultures that men should be more authoritative and mothers more compassionate. By looking at both parents from different cultural backgrounds, we may have a better understanding of how children are raised in different family situations [3].

A study in Jordan looked at the parental style of parents among children with an average range of 15 years. The study showed that most parents were practicing authoritative parenting, meaning that children had clear rules from supportive parents. The study showed that teens achieved better outcomes when one parent was authoritative, even if the other was not [4]. Previous research conducted in Saudi Arabia on parental style include a 2015 study which tested the hypothesis that abuse is a predictor of negative effects, such as negative emotions and reduced well-being, while encouragement is a predictor of positive effects, such as positive emotions and overall quality of life. In addition, the study aimed to validate a new tool for assessing parenting behaviors in Arab populations, specifically in Saudi Arabia. The results showed that even mild forms of abusive parenting had long-term effects on well-being into adulthood. This suggests that even parenting styles not typically labeled as “abusive” can have harmful effects [5]. Another study aimed to determine the prevalence of parenting styles among parents of adolescent children in Qassim, Saudi Arabia, in 2021. A total of 496 parents participated in this community-based, descriptive cross-sectional study. Data were collected using a structured questionnaire, including the Parenting Style Dimension Questionnaire (PSDQ) to identify each participant’s parenting style. The findings indicated that among younger parents, the authoritative parenting style was the most prevalent. In addition, the husband’s income and the size of the family were also factors that influenced the parents’ style. It has been suggested that creating parenting education programs is crucial to providing parents with the knowledge and skills they need to raise their children [6].

The main cause of dental caries is inadequate nutrition, particularly the consumption of frequent meals high in sugar [7]. Dental health is greatly influenced by food decisions, particularly sugar consumption, but maintaining good oral hygiene is crucial to avoiding tooth decay [8]. In 2024, a meta-analysis was conducted to determine the prevalence of dental caries in Saudi Arabia. It looked at dental caries in primary and permanent teeth in children aged 2–18 years. The average prevalence was 75.43% for primary teeth, and the average prevalence for permanent teeth was 67.7% [9]. Several studies have attempted to assess the relationship between parenting style and children’s oral health. In 2019, a study aimed to assess parents’ parenting style and their children’s oral health in Saudi Arabia. The study included 280 preschool children who had never visited a dentist. The two main parenting styles detected were authoritative and permissive. The study found no evidence of a relationship between parenting style and child oral hygiene and dental caries. However, the study found a slight correlation between parenting style and child’s dietary behaviors [10]. In 2020, a study conducted in Riyadh, Saudi Arabia, aimed to link parenting approaches with the dental behavior of their children. In their first dental appointment, 282 healthy preschoolers between the ages of three and six were enrolled in the pediatric dentistry clinic at KSU in Riyadh, Saudi Arabia. The parents or primary caregivers filled out the Parenting Styles and Dimensions Questionnaire. Two parenting styles were identified among Saudi parents in the KSU sample: permissive and authoritative. Parenting style affected a child’s dental behavior and was linked to parental dental anxiety [11].

There is a lack of research on parenting styles among Saudi Arabian parents in Riyadh. Examining and evaluating these aspects will help develop evidence-based strategies to improve parenting practices. The aim of this study is to identify the predominant parenting style among parents of primary school children and explore its association with children’s dental health The objectives are: (a) to identify the most common parenting style among parents of school-aged children, (b) to explore the association between the predominant parenting style and the prevalence of children’s dental caries, (c) to assess the association between the predominant parenting style and the frequency of tooth brushing, (d) to examine the association between the predominant parenting style and children’s dental visit patterns, and (e) to assess the association between the predominant parenting style and fruit consumption per day by the child.

## 2. Methodology

### 2.1. Study Design, Setting and Participants

This study is a cross-sectional study that assessed parents’ parenting style for children in public primary school in Riyadh, Kingdom of Saudi Arabia. A list of all governmental primary schools was obtained from the Ministry of Education. Fourteen schools representing five geographical regions were selected using a stratified random sampling within the framework of a related study conducted as part of the same research project [12].

**Inclusion criteria:**

- Parents with children in 1st, 2nd, 3rd grade (6–9 years).

**Exclusion criteria:**

- Parents whose children had chronic health conditions.
- Parents whose children were on long-term medications, such as those that include sugar or are known to decrease salivary flow.

### 2.2. Data Collection

**Parenting Style Assessment**

In their children’s bags, each of the eligible parents were given an information sheet, a consent form, and a structured parenting style questionnaire. Parents who had not returned their consent form and questionnaire (if they had misplaced them, an additional copy was sent) received a reminder email within a week, and those who had consented to participate received an acknowledgement. Parents were not required to participate and could choose not to at any time without providing a reason. The parenting style questionnaire is a validated and widely used instrument in parenting research [13]. To guarantee clarity, it was translated into Arabic, examined by a specialist in academia, and tested on 20 parents who were not involved in the study. Parenting style scores were calculated as the means of selected items corresponding to each style:

- Authoritative: items 1, 3, 5, 7, 9, 11, 12, 14, 18, 21, 22, 25, 27, 29, 31 (15 items)
- Authoritarian: items 2, 4, 6, 10, 13, 16, 19, 23, 26, 28, 30, 32 (12 items)
- Permissive: items 8, 15, 17, 20, 24 (5 items)

Higher mean scores indicate a stronger tendency toward the corresponding parenting style.

**Oral Health Assessment**

The collection of data on dental caries and children’s oral health was conducted in previous research [12] and is used in this paper to explore the association between parenting style and the prevalence of children’s dental caries, the frequency of fruit consumption, the frequency of tooth brushing and routine dental checkups. Data collection was performed by trained researchers as described in the related study. Calibration procedures, including assessment of inter- and intra-examiner reliability, were conducted prior to data collection to ensure measurement consistency [12]. Clinical examination for the prevalence of children’s dental caries was performed according to WHO criteria, that define dental caries at the caries into dentine threshold [14]. No radiographs were taken. Examinations were performed in empty classrooms; each child was examined one at a time and only the examiner and recorder were present during examination. Children were seated on a chair, and the headlamps, disposable mouth mirror and tongue depressors were used for dental assessment. Standard infection control was in place. For a child’s oral health, the WHO Oral Health Questionnaire for Children was used [14].

### 2.3. Data Storage and Analysis

Data were stored on University of Manchester-approved and securely backed-up servers, in encrypted files or folders. SPSS 29.01 was used for data analysis. Categorical variables were described using counts and percentages, while means and standard deviations [SD] were calculated for continuous variables. Chi-square and nonparametric tests were carried out to assess associations between parenting style and demographic factors. In addition, binary logistic regression, ordinal regression, and multinomial regression were applied to assess the association of the predominant parenting style on child health-related outcomes including dental caries prevalence, brushing frequency, fruit consumption, and routine dental check-ups.

### 2.4. Ethical Considerations

The consent form was provided, and a reminder email was sent by headteachers to encourage and ensure participation. Participants were informed that their involvement was voluntary, and they could withdraw from the study at any point without providing a reason. To ensure confidentiality, each participant was assigned a unique identification number during the recruitment process. These ID numbers were used throughout the study for data management purposes, replacing any personally identifiable information. Anonymized data were included in publications and conference presentations.

All necessary ethical approvals were in place. This study obtained approval from the University Research Ethics Committee (UREC) (Ref. No. 2024-16282-32966), the Institutional Review board at King Saud University (Ref. No. 23/0425/IRB), and the Ministry of Education in Saudi Arabia to both obtain a list of schools and access schools in Riyadh city (Ref. No. 4500028581). The study was conducted in accordance with ethical standards and the Declaration of Helsinki.

## 3. Results

This study aims to examine the parenting styles of parents in Riyadh, KSA. A total of 840 parents were invited, of whom 506 consented to participate. Among the participating parents, females represented 59.7%, and males accounted for 40.3%. The average age of participating parents was 38.96 years (SD 6.09). The primary ethnicity was Arab, constituting 99.8%, while non-Arab individuals accounted for 0.2%. Approximately 37.4% of parents had completed secondary school, 53.6% had a bachelor’s degree, and postgraduate education was less common at 9.1%. Full-time employment stood at 52.8%, while unemployment was at 38.7%. Families with incomes ranging from 2500 to 5000 constituted 20.2%, and those with incomes between 10,000 and 15,000 accounted for 22.7% (Table 1).

The findings indicated that the predominant parenting style across the studied groups was authoritative, including 449 out of 506 (88.7%) mothers and 412 out of 506 (81.4%) fathers, highlighting that most parents tended to balance warmth and discipline. Less parents were authoritarian, with only 12 (2.4%) out of 506 mothers and no fathers, indicating parents as less controlling and demanding when interacting with their children (Table 2).

This study also examined whether parenting styles varied according to several demographic factors, including parent age, gender, education level, and employment status. A chi-square test of independence was conducted, revealing evidence of an association between parenting style and family income for both mothers (χ^2^ = 41.48, df = 8, *p* < 0.001) and fathers (χ^2^ = 13.86, df = 4, *p* = 0.008). However, insufficient evidence of an association was found between mothers’ parenting style and gender (χ^2^ = 5.23, df = 2, *p* = 0.073), and no evidence with education level (χ^2^ = 3.91, df = 4, *p* = 0.418), employment status (χ^2^ = 10.76, df = 8, *p* = 0.216), or number of adults in the household (χ^2^ = 7.39, df = 8, *p* = 0.496). Similarly, for fathers, insufficient evidence of an association was found between parenting style and gender (χ^2^ = 3.02, df = 1, *p* = 0.082), and no evidence of an association with education level (χ^2^ = 4.49, df = 2, *p* = 0.106), employment status (χ^2^ = 7.56, df = 4, *p* = 0.109), and number of adults in the household (χ^2^ = 5.61, df = 4, *p* = 0.230) (Table 3).

Additionally, nonparametric tests were conducted to assess the association between parenting styles and continuous variables, such as parental age and the number of children in the household. Using the Kruskal–Wallis test, there was no evidence of an association between the number of children in a household (H = 5.66, df = 2, *p* = 0.059). Since fathers had only two parenting styles, the Mann–Whitney U test was conducted, and insufficient evidence of an association was found (U = 5964.5, z = −1.70, *p* = 0.088) for parental age, and no evidence was found for the number of children in a household (U = 7131.0, z = −0.09, *p* = 0.931) (Table 4).

To further investigate the potential impacts of authoritative parenting style on child health-related outcomes, additional analyses were conducted. These outcomes included dental caries, brushing frequency, routine dental check-ups, and fruit consumption per day. Depending on the nature of the outcome, binary logistic regression, ordinal regression, or multinomial regression was applied.

According to the previous research [12], the prevalence of dental caries was 79.6%. Of the parents surveyed, 21.0% said that their children brushed their teeth twice a day, 47.3% reported that they brush once a day, and 31.7% reported that they brush less frequently than once a day. Around 14.8% of the children went for a routine checkup, 28.8% never went to the dentist, and 51.2% of children only attended if they experienced discomfort or problems with their teeth and gums. A percentage of 25.1% of children consumed fresh fruit daily and 42.4% of children consumed fruit many times a week. Using binary logistic regression, the results showed that mothers’ and fathers’ authoritative parenting style showed no evidence of an association with the presence of children’s dental caries (OR = 1.021, 95% CI: 0.79, 1.31, *p* = 0.871), (OR = 0.940, 95% CI: 0.71, 1.22, *p* = 0.653), respectively. Similarly, multinomial regression was conducted for the variable routine dental checkups; this study found no evidence of an association for mothers (RRR = 1.047, 95% CI: 0.642, 1.708, *p* = 0.855) or fathers (RRR = 0.995, 95% CI: 0.596, 1.661, *p* = 0.985). Furthermore, ordinal regression was used to assess the association between the children’s brushing frequency and the authoritative parenting style; the study also found no evidence of an association for mothers (OR = 1.06, 95% CI: 0.87, 1.29, *p* = 0.557) and fathers (OR = 1.01, 95% CI: 0.90, 1.34, *p* = 0.317). However, with ordinal regression evidence of an association was found between mothers’ authoritative parenting style and higher fruit consumption in children (OR = 1.27, 95% CI: 1.028, 1.570, *p* = 0.027), meaning fruit consumption increased with greater authoritative style. No evidence of an association was found with fathers (OR = 1.165, 95% CI: 0.944, 1.437, *p* = 0.152).

## 4. Discussion

In the present study, the authoritative parenting style was the most frequent among the participants that took part. This conclusion is in line with research conducted in Saudi Arabia in 2019 that looked at the parenting practices of preschool-aged children who had never been to a dentist. In that study, the Parenting Style and Dimensions Questionnaire [PSDQ] was used to determine parenting styles, while children’s dental caries were diagnosed based on World Health Organization (WHO) criteria, and oral hygiene was assessed using the Simplified Debris Index (DI-S). It is interesting that while authoritative parenting was common in our sample, the 2019 survey found authoritarian parenting to be the most prevalent, observed in 94% of Saudi parents [10]. A 2021 study in Qassim, Saudi Arabia, investigated the parenting practices of parents of teens in the same way. The research discovered that younger parents mostly used an authoritative style, and that the style of parenting was strongly linked to the parent’s income [10]. This corresponds with the present study, which also found a significant association between parental income (both mother and father) and parenting style.

No evidence of an association was found between parenting style and children’s dental caries in this study. This finding is consistent with the results of a recent study conducted in 2024 at the Pediatric Dentistry Clinic, Cairo University. There were 180 parent–child pairings in the study, and the PSDQ was also used to determine the parenting style. The authoritative approach was the most common (67.2%), and just like this study, there was no association between parenting style and dental caries [15]. These findings suggest that several factors may play a role in the development of dental caries, and parenting style alone may not be one of them [15]. Dietary habits, dental hygiene, and access to dental treatment may be more important factors. Our study did not find an association between parenting style and how often children brush their teeth, but other studies have. For instance, the study from Cairo University revealed a connection between the way parents raise children and how often they clean their teeth and how many main meals they eat each day [15]. This contrast highlights the complex and context-dependent nature of these associations. Furthermore, this study found no evidence of an association between parenting style and dental visits; this was in contrast with previous research that found an association [16,17]. Also, our results showed a notable variation in how much fruit children ate based on their parenting style. This was in line with a previous study conducted in 2020 that found that the authoritative parenting style is associated with healthier dietary intake among preschool children [18]. This suggests that parenting styles can still affect what children eat, even if they do not directly change how they brush their teeth.

### 4.1. Strengths

The Parenting Style and Dimensions Questionnaire (PSDQ) was used to make sure that the data on parenting styles was reliable and could be compared. Many researchers use this tool, and it has been proven to work. The study also looked at important social and economic factors, such as parental education and income, which are known to influence aspects of parenting behavior that might affect how parents raise their children. The study also looked at dental caries, how often children brush their teeth, and what they eat, enabling further understanding of factors affecting child practices.

### 4.2. Limitations

The study was conducted in a specific place; hence its findings cannot be applied to other communities with differing cultural, socioeconomic, or healthcare contexts. Since research is cross-sectional, it only provides a snapshot in time. As a result, studies have failed to show that there is a direct relationship between parenting styles and issues with dental habits. Parenting styles were assessed by self-administered questionnaires, which might have been impacted by social desirability bias. This occurs when parents express their ideal parenting practices instead of their actual behaviors. The information on the child’s food habits and how often they brushed was provided by their parents and might not be a perfect reflection of their actual practices.

### 4.3. Recommendations

Researchers should undertake long-term studies to find out how different parenting methods affect children’s dental health practices and outcomes in the future. This will provide evidence around causation instead of just association. Parenting seminars or programs that teach authoritative parenting tactics like encouraging, positive reinforcement, and making routines should be a part of any effort to improve children’s oral health. Such programs could be integrated into school- or community-based oral health initiatives. Nutrition counseling should focus on the complete family, highlighting how important parents are in their children’s food choices, since authoritative parenting is linked to children eating more fruit and vegetables.

## 5. Conclusions

The current study revealed that the authoritative parenting style was stated most often among participants. No evidence of an association was seen between parenting style and the experience of dental caries or brushing frequency in this study; however, a difference in fruit and vegetable consumption was observed, with higher intake associated with mothers’ authoritative parenting style.

## Figures and Tables

**Table 1:** Demographic Characteristics.

Parent Gender	Female	59.7%
	Male	40.3%
Parent Age	Mean SD	38.96 6.09
Relationship with child	Mother	58.9%
	Father	39.3%
	Grandparent	0.6%
	Guardian	1.2%
Ethnicity	Arab	99.8%
	Asian	0.2%
Educational Level	≤Secondary school	37.4%
	Bachelor	53.6%
	Postgraduate	9.1%
Employment Status	Full time employed	52.8%
	Self employed	3.0%
	Part time employed	4.7%
	Student	0.8%
	Unemployed	38.7%
Total Family Income	<2500	6.3%
	2500 ≥ 5000	20.2%
	5000 ≥ 10,000	32.2%
	10,000 ≥ 15,000	22.7%
	>15,000	18.6%

**Table 2:** Frequency of Reported Parenting Styles.

Mother’s Dominant Style	n	%
Authoritarian	12	2.4
Authoritative	449	88.7
Permissive	23	4.5
Tied	21	4.2
Missing	1	0.2
Father’s Dominant Style	n	%
Authoritative	412	81.4
Permissive	35	6.9
Tied	13	2.6
Missing	46	9.1

**Table 3:** Association between Parenting Style and Categorical Sociodemographic Factors.

Mother	Chi-Square (χ^2^)	df	*p* Value
Gender	5.230	2	0.073
Educational level	3.910	4	0.418
Employment status	10.760	8	0.216
Family income	41.676	8	*p* < 0.001 *
Number of adults in household	7.385	8	0.496
**Father**	**Chi-Square (χ^2^)**	**df**	***p*** ** Value**
Gender	3.017	1	0.082
Educational level	4.490	2	0.106
Employment status	7.556	4	0.109
Family income	13.862	4	0.008
Number of adults in household	5.613	4	0.230

* *p* < 0.05. df: degree of freedom

**Table 4:** Parenting Style Difference based on Continuous Sociodemographic Factors.

Independent Samples Kruskal–Wallis Test	
Mother	*p*-Value
The distribution of parent age is the same across categories of parental style	0.995
The distribution of the number of children in households is the same across categories of parental style	0.059
**Independent Samples Mann–Whitney U Test**	
**Father**	***p*** **-Value**
The distribution of parent age is the same across categories of parental style	0.088
The distribution of the number of children in households is the same across categories of parental style	0.931

## Data Availability

The data presented in this study are available on request from the corresponding author. The data are not publicly available due to privacy and ethical reasons.

## Abbreviations

PSDQ	Parenting Style Dimension Questionnaire
